# Pretreatment of Huoxue Jiedu Formula Ameliorates Myocardial Ischaemia/Reperfusion Injury by Decreasing Autophagy via Activation of the PI3K/AKT/mTOR Pathway

**DOI:** 10.3389/fphar.2021.608790

**Published:** 2021-02-26

**Authors:** Linzi Long, Zikai Yu, ShengJun Chen, Jiarui Wu, Yingying Liu, Jun Peng, Hua Qu, Changgeng Fu

**Affiliations:** ^1^ Academy of Integrative Medicine, Fujian University of Traditional Chinese Medicine, Fuzhou, China; ^2^ Xiyuan Hospital, China Academy of Chinese Medical Sciences, Beijing, China; ^3^ National Clinical Research Center for Chinese Medicine Cardiology, Xiyuan Hospital, China Academy of Chinese Medical Sciences, Beijing, China; ^4^ Jiangyin Tianjiang Pharmaceutical Co., Ltd., Jiangsu, China; ^5^ Department of Clinical Chinese Pharmacy, School of Chinese Materia Medica, Beijing University of Chinese Medicine, Beijing, China

**Keywords:** ischaemia/reperfusion, network pharmacology, Chinese herb, PI3K-AKT pathway, autophagy

## Abstract

**Background:** Myocardial ischaemia/reperfusion (I/R) results in myocardial injury via excessive autophagy. Huoxue Jiedu Formula (HJF) has been widely applied in China for the treatment of ischaemic heart disease. However, the mechanisms of HJF are still poorly understood. Thus, the present experiment was designed to observe the effects of HJF on myocardial I/R injury and explore the possible mechanism.

**Methods:** Myocardial injury in rats subjected to myocardial I/R was reflected by nitrotetrazolium blue chloride staining, thioflavin S staining, serum creatine kinase-MB (CK-MB) and cardiac troponin T (cTnT). Autophagy was determined by electron microscopy, laser confocal microscopy, Q-PCR and western blot. The possible pathway was predicted by network pharmacology and validated *in vivo* and *in vitro*.

**Results:** Pretreatment of HJF decreased the no-reflow area, infarcted area, serum CK-MB levels and serum cTnT levels in I/R rat model. In addition, pretreatment of HJF decreased autophagy in heart tissues (decrease in Beclin-1 and LC3-II, and increase in Bcl-2, p62 and ratio of LC3-I/LC3-II). In the vivo study, pretreatment of HJF significantly decreased hypoxia/reoxygenation (H/R)-induced autophagy in H9C2 cells. Network pharmacology was applied to predict the possible mechanism by which HJF affects cardiac autophagy, and the PI3K/AKT/mTOR signalling pathway was the most significantly enriched pathway. And experimental studies demonstrated that pretreatment of HJF increased the phosphorylation of AKT and mTOR, and the effects of HJF on autophagy would be offset by PI3K inhibitor LY294002.

**Conclusion:** Pretreatment of HJF ameliorates myocardial I/R injury by decreasing autophagy through activating PI3K/AKT/mTOR pathway.

## Introduction

Ischaemic heart disease is the main reason for morbidity and mortality in all over the world. Reperfusion therapy decreases the myocardial infarct area and increases cardiac function in the patients suffered from acute myocardial infarction; whereas reperfusion exacerbates myocardial injury, which is also called myocardial ischaemia/reperfusion (I/R) injury (Hausenloy and Yellon, 2015; Gewirtz and Dilsizian, 2017). Myocardial reperfusion induces oxidative stress and leads to cardiac autophagy (Seidlmayer et al., 2015; Bourke et al., 2018). Increasing evidences have shown that excessive autophagy is associated with myocardial I/R injury (Kini and Singh, 2007; Nishida et al., 2009). Excessive autophagy, which degrades organelles and proteins in cell, results in cardiomyocyte death (Zhu et al., 2007; Kroemer and Levine, 2008; Fan et al., 2016; Huang et al., 2017). Preventing the excessive autophagy in myocardial I/R would be a promising approach to alleviate cardiomyocyte death and conserve cardiac function. Our previous clinical study showed that Qing-Xin-Jie-Yu Granule (QXJYG) reduced the risks of adverse thrombotic events in the patients with stable coronary disease (Li et al., 2019).

Huoxue Jiedu Formula (HJF), simplified form of QXJYG and consisting of *Paeonia lactiflora* Pall, *Ligusticum striatum* DC and *Coptis teeta* Wall, is widely used in China to treat ischaemic heart disease. Previous studies demonstrated that the components of HJF, such as paeoniflorin, berberine and tetramethylpyrazine, exhibited cardiovascular protective effects by regulating autophagy (Fan et al., 2015; Wen et al., 2019; Xu et al., 2020). In the current study, we evaluated effects of HJF on I/R-induced myocardial injury and explored the possible mechanism.

## Materials and Methods

### Ethics Statement

The animal experiments were performed according to the ethical guidelines of US National Institutes of Health Guide for the Care and Use of Laboratory Animals. And the protocols of the experiments associated with animal were approved by the Institutional Animal Care and Use Committee of Xiyuan Hospital of the China Academy of Chinese Medical Sciences (Beijing, China).

### Animals

Male Wistar rats, with the age of 8 weeks and weight of 200 ± 20 g, were obtained from Changzhou Cavens Bioscience Co., Ltd. (Changzhou, Jiangsu, China). And the rats were randomly assigned to the Sham group (n = 16), ischaemia/reperfusion (I/R) group (n = 16), low-dose HJF group (n = 16), middle-dose HJF group (n = 16), high-dose HJF group (n = 16) and aspirin group (n = 16). The rats lived in the condition of specific pathogen-free, with a temperature of 22°C and a 12-h light/dark cycle, and the rats were free to eat food and drink water.

### Cell Culture

H9C2 cell line was obtained from Tongpai Shanghai Biotechnology Co., Ltd. (Shanghai, China). The cells were cultured in Dulbecco’s Modified Eagle Medium (DMEM, Gibco, 31985-070, Germany), containing 10% Fetal Bovine Serum (FBS) and antibiotics (100 units/mL penicillin and 100 mg/ml streptomycin) at 37°C in humidified air with 5% CO_2_.

### Drug Administration

HJF consists of the extracts of *Paeonia lactiflora* Pall (PLP, Jiangyin Tianjiang Pharmaceutical Co., Ltd., No.11712723), *Ligusticum striatum* DC (LS, Jiangyin Tianjiang Pharmaceutical Co., Ltd., No.11803801) and *Coptis teeta* Wall (CTW, Jiangyin Tianjiang Pharmaceutical Co., Ltd., No.11705806). The mixture was combined with the extracts of PLP, LS and LC at a 1:1:1 ratio. The drugs were dissolved in 0.9% saline solution (NS) and stored at 4°C. The rats were allowed to acclimate for 1 week prior to the experiments. The dose of HJF administrated to rat was calculated based on body surface area (Nair and Jacob, 2016). The rats in the group of low-dose were pretreated with the dose of 270 mg/kg/day, the rats in the group of middle-dose were pretreated with the dose of 540 mg/kg/day, and the rats in the group of high-dose were pretreated with the dose of 1,080 mg/kg/day. The rats in the Sham group and I/R group received an equivalent volume of 0.9% NS solution. The rats in the positive control group were pretreated with aspirin (30 mg/kg/day). For the cell culture experiments, the stock solution of the drug was freshly prepared by dissolving the drug in PBS to the desired concentration and filtering.

### Cell Counting Kit-8 Assay

H9C2 cells at log-phase were seeded in 96-well plates at a density of 0.5 × 10^5^ cells per well in 100 µL medium and incubated at 37°C in 5% CO_2_. When the cells reached 40∼50%, they would be starved in serum-free media. Then, the cells were incubated with different concentrations of HJF (0.01, 0.05, 0.10, 0.15, 0.3, 0.6, 1.2, or 2.4 mg/ml) for 24 h, then 10 µL CCK-8 reagent (Beyotime, No. C0037) was added to each well and incubated for 4 h at 37°C. The optical density (OD) at 450 nm was measured with a microplate reader (Thermo, MULTISKAN MK3). Cell viability was determined using the following formula: Cell viability was calculated by according to the ratio of absorbance of the experimental samples and the control samples. The optimal concentration of the drug was 0.15 mg/ml, and we chose this concentration for the subsequent autophagy mechanism study.

### I/R Injury Animal Model and Sample Collection

After 14 days of treatment, the myocardial I/R model was established as follows (Wu et al., 2018). The rats were anaesthetized with 3.5% chloral hydrate at a dose of 10 ml/kg via intraperitoneal injection. After the rats were anaesthetized, under a sterile environment, the heart was exposed from a small left anterior thoracotomy. The blood vessel under the left anterior descending branch of the coronary artery (2 mm) was bluntly separated. A soft silicone tube with a length of approximately 0.5 to 1 cm was placed between the blood vessel and the ligation line (No. 0 surgical line), and then, the coronary artery was ligated with the No. 0 surgical line. The heart was immediately returned to the chest cavity. The cardiac I/R injury rat model was established by ligaturing for 90 min, followed by reperfusion for 2 h. The rats were immediately sacrificed when the experiment ended, and arterial blood and heart samples were extracted after 2 h of reperfusion.

### Measurement of Serum Creatine Kinase-MB and Cardiac Troponin T Levels

The blood was collected from abdominal aorta and the serum was separated, then the levels of creatine kinase-MB (CK-MB) and cardiac troponin T (cTnT) were measured by colorimetric method using an automatic biochemical analyser (Olympus AU-400).

### Nitrotetrazolium Blue Chloride Staining

After reperfusion, three rats were randomly selected from each group, and the hearts of the rats were put in 0.2% nitrotetrazolium blue chloride (N-BT) staining solution and gradually stained at room temperature for 2 to 3 min. The front and back sides of the myocardium were scanned to obtain images, and a multimedia color pathological image analysis system was used to measure the infarct area on each side of each myocardium. The infarcted area was grey-white, and the non-infarcted area was blue-black. We calculated the percentage of the infarct area to the whole heart area.

### Thioflavin S Staining

After reperfusion, three rats were randomly selected from each group, and 6% thioflavin S (1 ml/kg) was injected into the femoral vein. The rats were immediately sacrificed when the drug entered the right atrium with blood circulation. The heart was quickly removed. The ventricle was partially cut into pieces and weighed separately, and the heart slices were placed in the dark box of the thin layer chromatograph. The fluorescence was observed under a 365-nm wavelength light source. Both sides of each section were digitally photographed by a multimedia color pathological image analysis system. For each myocardium, the non-reflow zone indicated that sulfurin was not colored and dark black, and the reflow zone showed that sulfurin was colored and bright blue-green. We calculated the percentage of the whole heart area without a reflow zone.

### RNA Extraction and Q-PCR

The LC3, Beclin-1, and Bcl-2 gene mRNA expression levels were quantified using Q-PCR. Briefly, we collected rat myocardial tissue without reflow or H9C2 cells, and 100 mg of myocardial tissue was lysed to extract RNA with Trizol reagent (Aidlab, Lot: 252250AX). The extracted RNA was measured by the ratio of the absorbance at 260 nm to that at 280 nm with a microspectrophotometer (Hangzhou ALL SHENG Instrument Co., Ltd., Nano-100, China). And Q-PCR was performed using SYBR Green Master Mix (Vazyme, Naijing, China, No. Q111-02) with a real-time fluorescence quantitative PCR instrument (ABI, QuantStudio 6, United States). The relative mRNA expression were normalized according to GAPDH and quantified with the formula 2-ΔΔCT. The primer sequences are as follows:

GAPDH: Forward, 5′- TGT​GGG​CAT​CAA​TGG​ATT​TGG -3′ and Reverse, 5′- ACA​CCA​TGT​ATT​CCG​GGT​CAA​T-3’;

LC3: Forward, 5′- CTT​CTG​AGC​CAG​CAG​TAG​GG-3′ and Reverse, 5′-GAG​GGA​CAA​CCC​TAA​CAC​GA-3’;

Beclin-1: Forward, 5′-AGG​TTG​AGA​AAG​GCG​AGA​CA-3′ and Reverse, 5′-AAT​TGT​GAG​GAC​ACC​CAA​GC-3’;

Bcl2: Forward, 5′-GAG​GAT​TGT​GGC​CTT​CTT​TG-3′ and Reverse, 5′-ACA​GTT​CCA​CAA​AGG​CAT​CC-3’.

### Protein Isolation and Western Blot Analysis

Myocardial tissue without reflow or H9C2 cells was lyzed with RIPA lysis buffer on ice for 20 min. The concentrations of protein were quantified with a BCA assay, and each sample was denatured. Equal amounts of total protein were resolved by polyacrylamide gel electrophoresis and transferred onto Poly vinylidene fluoride (PVDF) membranes. The membranes were blocked for 2 h with 5% bovine serum albumin. Then, the membranes were incubated with primary antibodies against Beclin-1 (Abcam, No. ab62557), LC3 (CST, No. 12741), AKT (Affinity, No. AF6261), *p*-AKT (Affinity, No. AF0016), mTOR (Affinity, No. AF6308), *p*-mTOR (Affinity, No. AF3308), p65 (CST, No. 4764), p-p65 (CST, No. 3031) and GAPDH (Abcam, No. ab8245) (all at 1:1,000 dilutions) overnight at 4°C followed by incubation with an HRP-conjugated secondary antibody (1:5,000 dilution) at room temperature for 1 h. The western blot images were acquired using an enhanced chemiluminescence system on Hyperfilm X-ray films (Beijing JUNYI Electrophoresis Co., Ltd., JY02S). Image Lab software was used for densitometric analysis. The protein level of GAPDH was used as the loading control.

### Observation of Autophagic Vesicles

H9C2 cells were pretreated with the drugs for 24 h and underwent hypoxia-reoxygenation intervention. The cells were digested with 0.25% trypsin and collected by centrifugation. The cells were fixed with glutaraldehyde and osmium acid, dehydrated with various concentrations of alcohol, impregnated, embedded in resin, and cut into thick sections. The sections were stained with 3% uranium acetate-lead citrate and observed using a transmission electron microscope.

### Observation of Autophagic Flux

H9C2 cells at log-phase were seeded in 24-well plates at a suitable cell density. When the cell confluence reached 80–90%, a plasmid expressing GFP-labelled LC3 was transfected into the H9C2 cells. After 24 h of incubation, the cells were treated in groups and stained with DAPI. After treatment, the cells of each group were photographed under confocal microscopy to analyze the autophagic flux.

### Identification of the Components of Huoxue Jiedu Formula

To prepare the HJF sample solution, 0.2 g PLP powder was precisely weighed and extracted with 50 ml methanol-hydrochloric acid (100:1 v/v) under ultrasonication (40 kHz) for 45 min. After this process, 1 ml solution was diluted with 9 ml hydrochloric acid. Then, 20 μL of the above solution was injected into UPLC-Q-TOF/MS system (Agilent G6530 Accurate-Mass Q-TOF) for analysis. LS powder (0.1 g) was precisely weighed and extracted with 50 ml ethanol under ultrasonication (40 kHz) for 30 min. Then, 1 μL of the above solution was injected into the UPLC-Q-TOF/MS system (Agilent G6530 Accurate-Mass Q-TOF) for analysis. CTW powder (0.2 g) was precisely weighed and extracted with 50 ml methanol-hydrochloric acid (100:1 v/v) under ultrasonication (40 kHz) for 45 min. After this process, 1 ml solution was diluted with 9 ml hydrochloric acid. One microlitre of the above solution was injected into the UPLC-Q-TOF/MS system (Agilent G6530 Accurate-Mass Q-TOF) for analysis. The UPLC-Q-TOF/MS system was set as a dual-jet ESI ion source, with a drying temperature at 300°C, flow rate of 8 L/min, and atomizer pressure of 35 psi. The capillary voltage in the positive ion mode was 4000 V, the negative ion mode was 3500 V, the capillary outlet voltage was 175 V, and the taper hole voltage was 65 V. The high-resolution mode was used for data acquisition with a mass/charge ratio of M/Z100∼2000. The sampling speed was 1 spectra/s, and the sampling time was 1,000 ms/spectra. TFANH4 anion (112.985587) and HP-0921 anion (1033.988109) were used. The positive ions were calibrated in real time using purine (111.050873) and HP-0921 positive ions (922.009798), with an atomization pressure of 5 psi.

### Predicting Functional Targets of Huoxue Jiedu Formula and Identifying Cardiac Autophagy-Related Genes

The identified components of HJF were searched in Swiss Target Prediction (http://www.swisstargetprediction.ch/) (Daina et al., 2019). SwissTargetPrediction predicts targets of small molecules according to similarity principle. The structure of molecules, in 2D or 3D, is used to predict from 3,068 macromolecular targets. Cardiac autophagy-related genes were collected from GeneCards (with Relevance Score ≥2) (https://auth.lifemapsc.com/) and Online Mendelian Inheritance in Man (OMIM) (http://omim.org/). The search terms included “cardiac autophagy or myocardial autophagy.”

### Statistical Analysis

Quantitative data data were present as mean ± standard deviation (SD) and analyzed with SPSS software (Version 22.0). Paired or independent Student’s t-test was performed to determine statistical significance between two groups, and one-way analysis of variance was used among ≥3 groups. *p* value <0.05 was considered statistically significant.

## Results

### Pretreatment of Huoxue Jiedu Formula Decreased Myocardial Injury Induced by I/R in Rats

**FIGURE 1:**
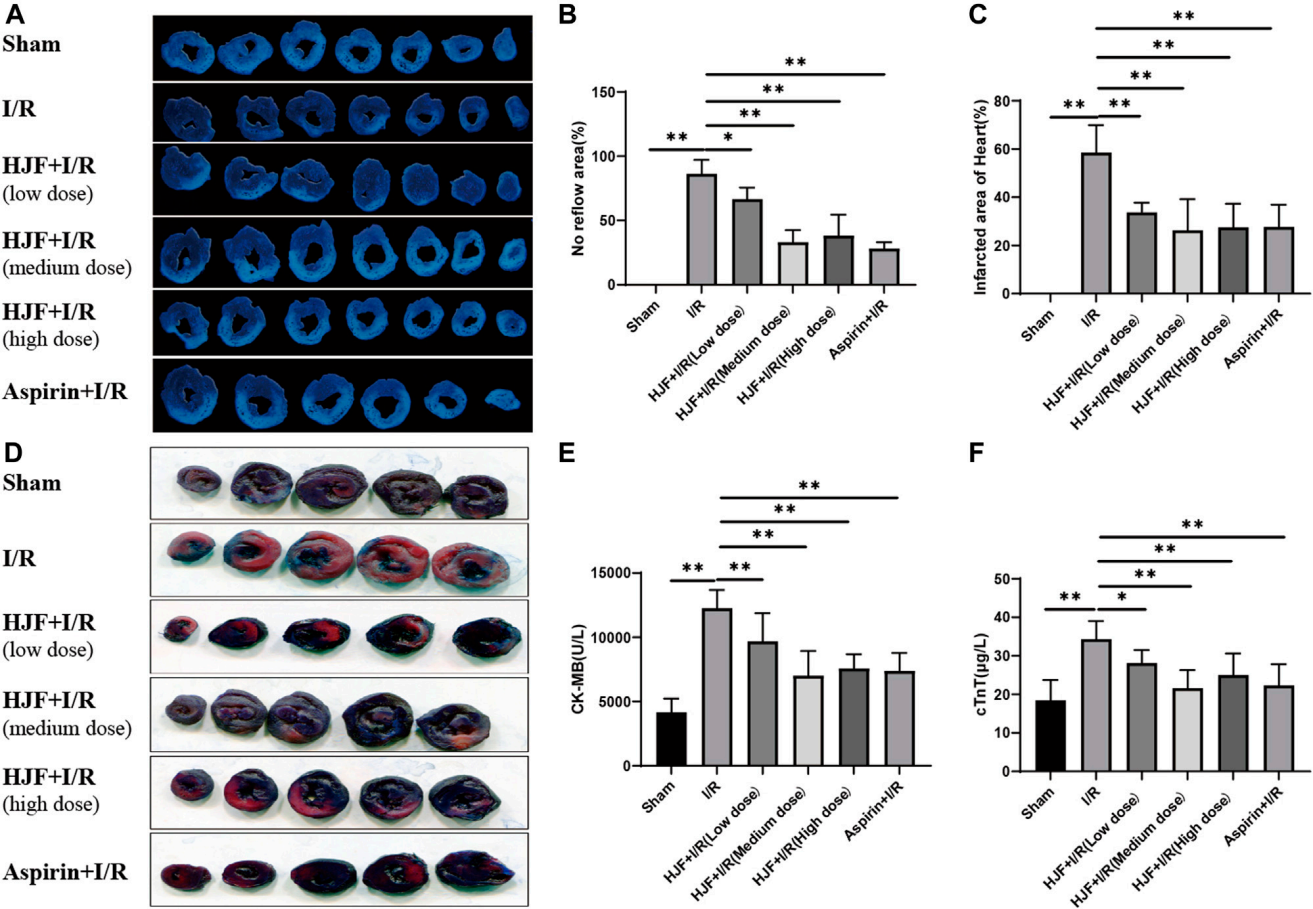
HJF treatment decreased myocardial injury induced by I/R in rats. **(A)**, Representative images of nitrotetrazolium blue chloride staining of the heart; **(B)**, No reflow area of the heart (n = 3); **(C)**, Infarcted area of the heart (n = 3); **(D)**, Representative images of thioflavin S staining of the heart; **(E)**, Serum level of CK-MB (n = 6); **(F)**, Serum level of cTnT (n = 6). The data are shown as the means ± SDs in each group. *p < 0.05, **p < 0.01. I/R, ischaemia/reperfusion; HJF, Huoxue Jiedu Formula; CK-MB, creatine kinase isoenzyme MB; c-TnT, cardiac troponin T.

To observe the myocardial injury induced by myocardial I/R, thioflavin S staining and N-BT chloride staining were performed on rat hearts, and the serum CK-MB and cTnT levels were detected. Compared with the sham group, the I/R group exhibited increased no-reflow area, infarcted area, and serum CK-MB and cTnT levels (Figure 1). Compared with the I/R group, HJF groups (270, 540, and 1,080 mg/kg) exhibited significantly decreased no-reflow areas and infarcted areas in the heart (*p* < 0.05) (Figures 1A–D). In addition, pretreatment of HJF (270, 540, and 1,080 mg/kg) also obviously decreased the serum CK-MB and cTnT levels compared with I/R group, as shown in Figures 1E,F.

### Pretreatment of Huoxue Jiedu Formula Decreased Cardiac Autophagy Induced by I/R in Rats

**FIGURE 2:**
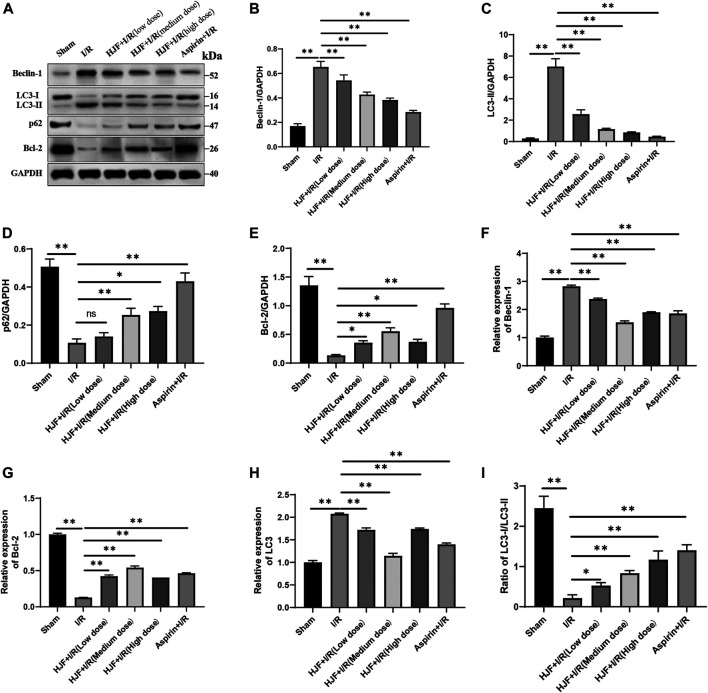
Effects of HJF on autophagy-related proteins in rats subjected to I/R. **(A)**, Representative western blot images of autophagy-related proteins; **(B)**, Quantification of the levels of Beclin-1; **(C)**, Quantification of the levels of LC3-II; **(D)**, Quantification of the levels of p62; **(E)**, Quantification of the levels of Bcl-2; **(F)**, mRNA expression of Beclin-1; **(G)**, mRNA expression of Bcl-2; **(H)**, mRNA expression of LC3; **(I)**, Ratio of LC3-I/LC3-II. The data are shown as the means ± SDs in each group (n = 3). *p < 0.05, **p < 0.01. I/R, ischaemia/reperfusion; HJF, Huoxue Jiedu Formula.

Markers of autophagy **(**Beclin-1, Bcl-2, LC3, and p62) were detected to explore the effects of HJF on the cardiac autophagy induced by I/R. The results of western blot demonstrated that compared with the control group, the I/R group exhibited the increased levels of Beclin-1 and LC3-II and the decreased levels of Bcl-2, p62 and ratio of LC3-I/LC3-II in the rat heart tissues (Figures 2A–E, I, ). Compared with the I/R group, the HJF group exhibited significant downregulation of Beclin-1 and LC3-II and upregulation of Bcl-2, p62 and ratio of LC3-I/LC3-II (Figures 2A–E, I). The Q-PCR results also demonstrated that I/R intervention increased the levels of Beclin-1 and LC3 and decreased the level of Bcl-2; however, pretreatment of HJF (270, 540, and 1,080 mg/kg) effectively reversed this trend (Figures 2F–H). All of the above data suggested that pretreatment of HJF could decrease the autophagy induced by I/R.

### Pretreatment of Huoxue Jiedu Formula Decreased the H/R-Induced Autophagy in H9C2 Cells

**FIGURE 3:**
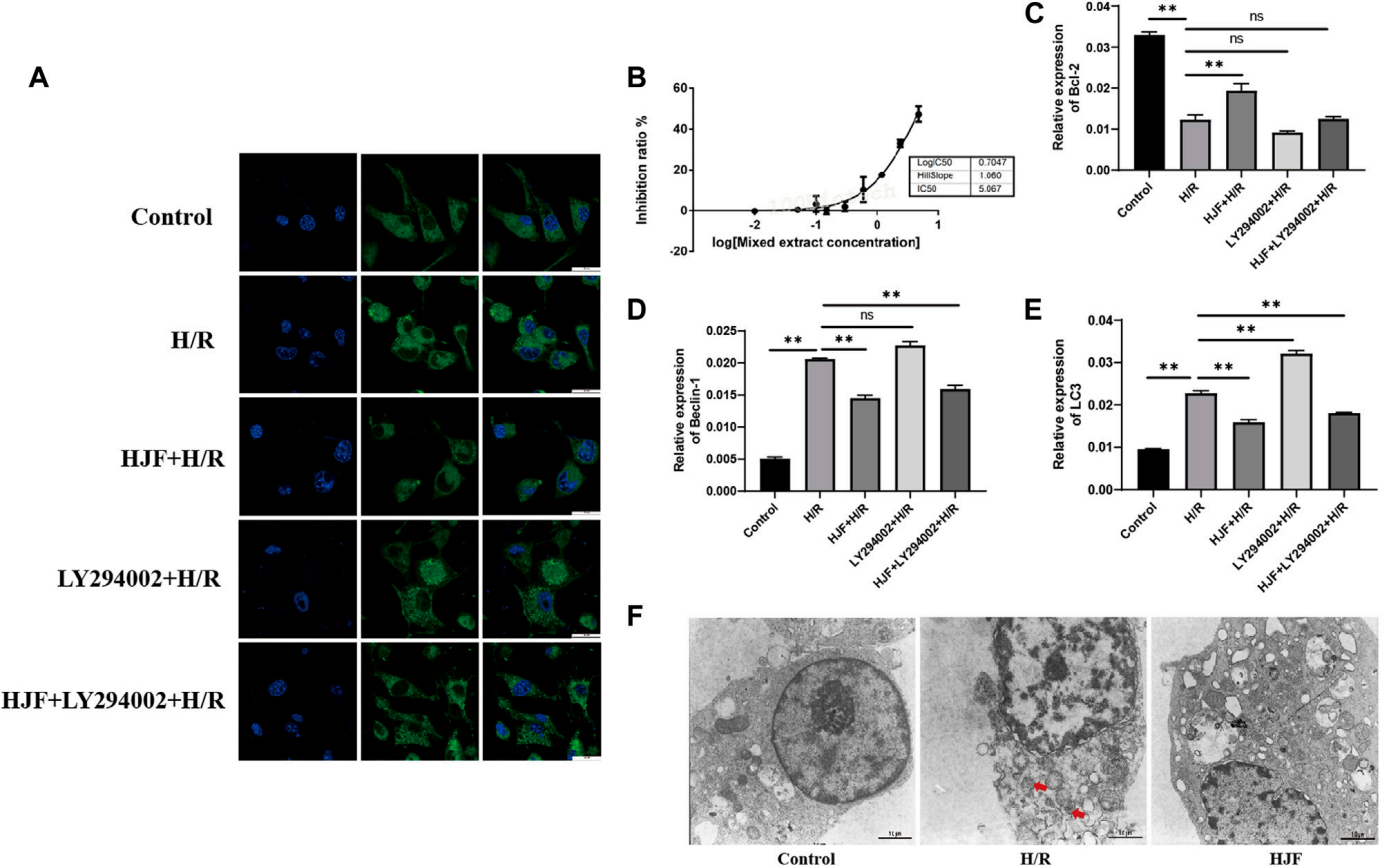
Effects of HJF on H/R-induced autophagy in H9C2 cells. **(A)**, Images of LC3 under laser confocal microscopy with a scaleplate of 25 μm (n = 3); **(B)**, IC50 of HJF; **(C)**, mRNA expression of Bcl-1 (n = 6); **(D)**, mRNA expression of Beclin-1 (n = 6); **(E)**, mRNA expression of LC3 (n = 6); **(F)**, Autophagosome formation observed by transmission electron microscopy with a scaleplate of 1 μm (marked with blue arrow) (n = 3). The data are shown as the means ± SDs in each group. *p < 0.05, **p < 0.01. H/R, Hypoxia/reoxygenation; HJF, Huoxue Jiedu Formula.

**FIGURE 4:**
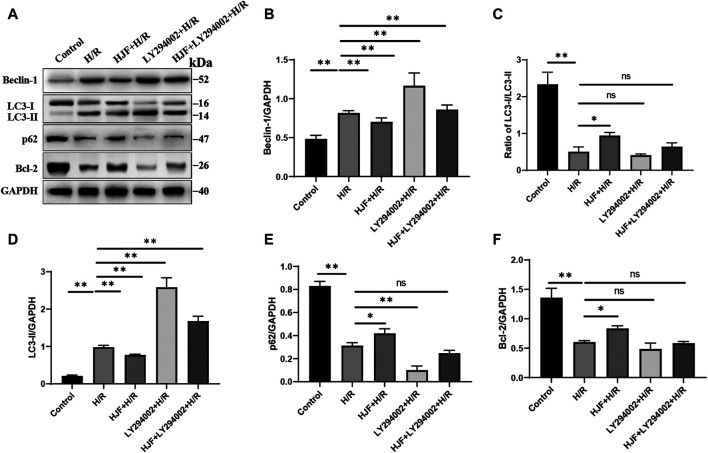
Effects of HJF on autophagy-related proteins in H9C2 cells exposed to H/R. **(A)**, Representative western blot images of autophagy-related proteins; **(B)**, Quantification of the levels of Beclin-1; **(C)**, Quantification of the levels of LC3-II; **(D)**, Ratio of LC3-I/LC3-II. **(E)**, Quantification of the levels of p62; **(F)**, Quantification of the levels of Bcl-2. The data are shown as the means ± SDs in each group (n = 3). *p < 0.05, **p < 0.01. H/R, Hypoxia/reoxygenation; HJF, Huoxue Jiedu Formula.

H9C2 cells were treated with different concentrations of HJF (0.01, 0.05, 0.10, 0.15, 0.3, 0.6, 1.2, and 2.4 mg/ml), and cell viability was measured with CCK-8 assays. The results showed that the IC_50_ of HJF was 5.067 mg/ml, and HJF at a concentration of 0.15 mg/ml showed no notable effect on H9C2 cell growth; therefore, this concentration was chosen for further cellular experiments (Figure 3B). Laser confocal microscopy and transmission electron microscopy were used to observe the autophagic flux and autophagosome formation in H9C2 cells, and the results demonstrated that the autophagic flux and autophagosome formation was increased in the H/R group compared with the control group. Compared with the H/R group, the HJF group (0.15 mg/ml) exhibited decreased autophagic flux and autophagosome formation (Figures 3A, F). In addition, markers of autophagy **(**Beclin-1, Bcl-2, LC3, and p62) were detected to explore the effects of HJF on the H/R-induced autophagy in H9C2 cells. The Q-PCR results demonstrated that exposure to H/R increased the levels of Beclin-1 and LC3 and decreased the levels of Bcl-2, while pretreatment of HJF (0.15 mg/ml) effectively reversed this trend (Figures 3C–E). The western blot results demonstrated that compared with those in the control group, the H9C2 cells in the H/R group exhibited the increased levels of Beclin-1 and LC3 and the decreased levels of Bcl-2, p62 and ratio of LC3-I/LC3-II (Figure 4). Compared with the H/R group, the HJF group exhibited significant downregulation of Beclin-1 and LC3 and upregulation of Bcl-2, p62 and ratio of LC3-I/LC3-II (Figure 4).

### The PI3K/AKT/mTOR Signalling Pathway was Predicted by Network Pharmacology

**FIGURE 5:**
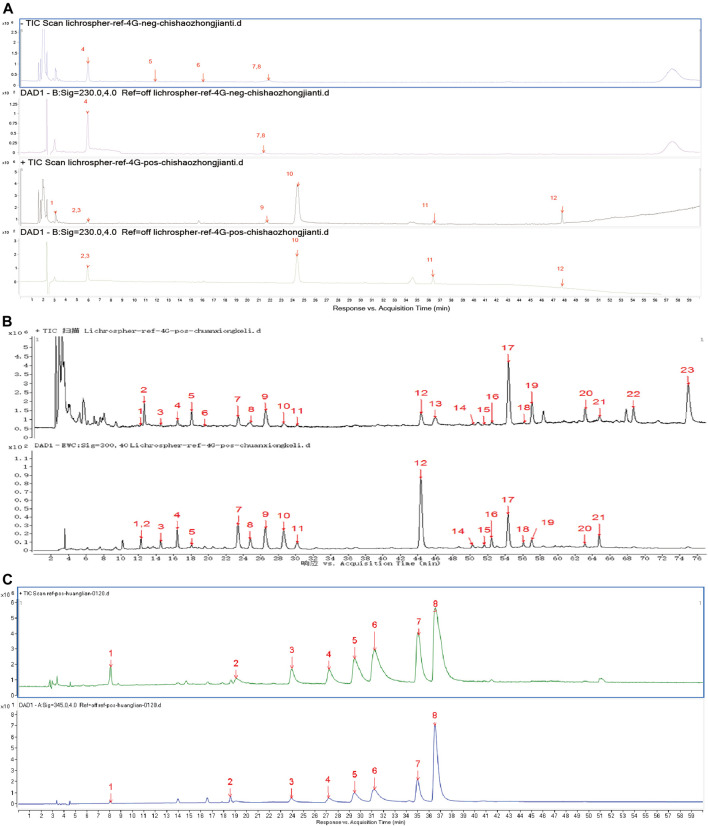
The components of HJF identified from UPLC-Q-TOF/MS. **(A)**, Twelve components identified in *Radix Paeoniae Rubra*: 8-debenzoylpaeoniflorin, gallic acid, 1-O-β-D-glucopyranosyl-paeonisuffrone, galloylsucrose, mudanpioside F, anthocyanidin B7, oxypaeoniflorin, cianidanol, albiflorin, paeonioflorin, pentagalloylglucose and 4″-hydroxy-6′-benzoylpaeoniflorin; **(B)**, twenty-three components identified in *Ligusticum wallichii*: 5-hydroxymethylfurfural, phenylalanine, isomer of chlorogenic acid, neochlorogenic acid, tryptophan, *p*-hydroxybenzoic acid, chlorogenic acid, cryptochlorogenic acid, isovanillic acid, caffeic acid, cynarin, ferulic acid, senkyunolide J, cis-1,5-dicaffeoylquinic acid, isochlorogenic acid B, isochlorogenic acid A, senkyunolide I, isochlorogenic acid C, senkyunolide H, 3-butylidenephthalide, proantho cyanidins, senkyunolide G and senkyunolide A; **(C)**, eight components identified in *Rhizoma coptidiswas*: magnoflorine, groenlandicine, columbamine, jatrorrhizine, epiberberine, coptisine, palmatine and berberine.

According to the UPLC-Q-TOF/MS results, we identified 12 compounds in the extracts of PLP (Figure 5A), 23 compounds in the extracts of LS (Figure 5B), and eight compounds in the extracts of CTW (Figure 5C). Thirty-one components of HJF were used to predict its target genes based on the principle of chemical structure similarity, and 807 targets were found (Data sheet 1 in Supplementary Material). In addition, 1800 cardiac autophagy-related genes with Relevance Score ≥2 were found (Data sheet 2 in Supplementary Material). Finally, 302 coincident targets was picked out between the target genes of HJF and cardiac autophagy-related genes (Data sheet 3 in Supplementary Material).

**FIGURE 6:**
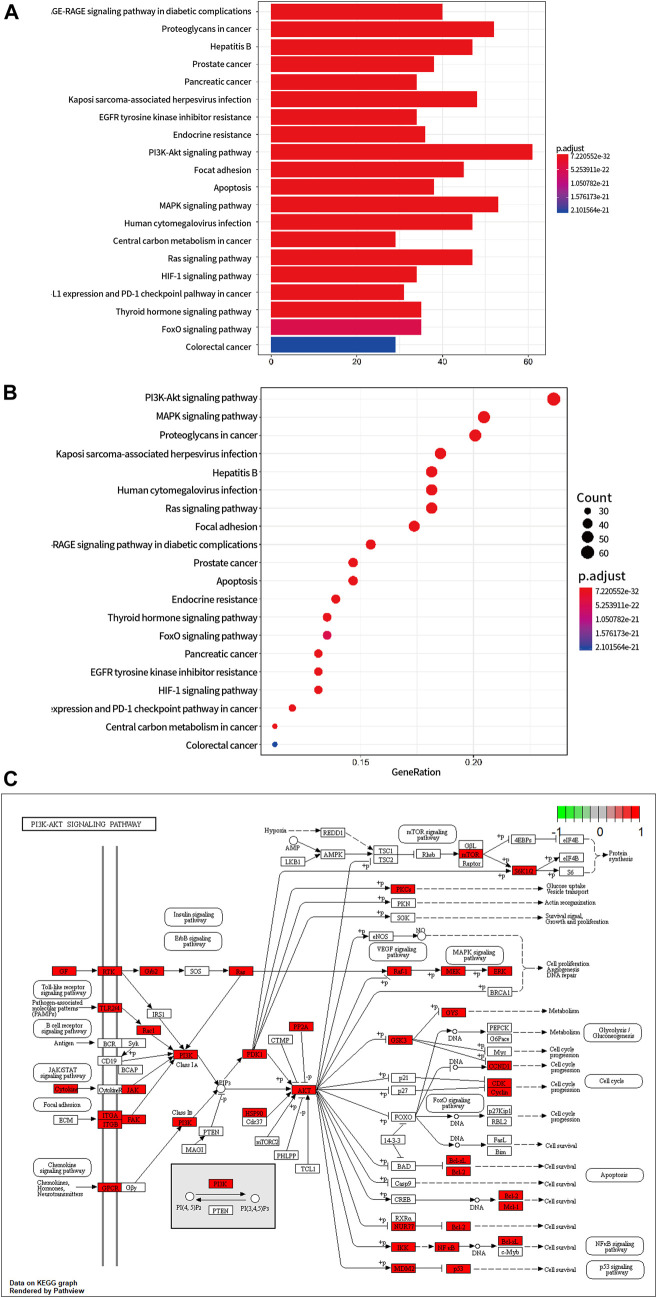
Prediction of the core signalling pathway by which HJF regulates cardiac autophagy. **(A)**, Bar graph of KEGG enrichment analysis; **(B)**, Bubble chart of KEGG enrichment analysis; **(C)**, PI3K-AKT signalling pathway; the red box represents the target genes of HJF.

To explore the possible pathway by which HJF regulating autophagy, we uploaded the 302 coincident targets into the DAVID database. Moreover, those terms were sorted according to their *p* values. According to KEGG enrichment analysis, the pharmacological effects of HJF on cardiac autophagy were associated with the PI3K-AKT signalling pathway, AGE-RAGE signalling pathway in diabetic complications, and so on. And PI3K-AKT signalling pathway was the most significantly enriched pathway (Figure 6).

### Pretreatment of Huoxue Jiedu Formula Alleviated Cardiac Autophagy by Activating PI3K/AKT/mTOR Signalling Pathway

**FIGURE 7:**
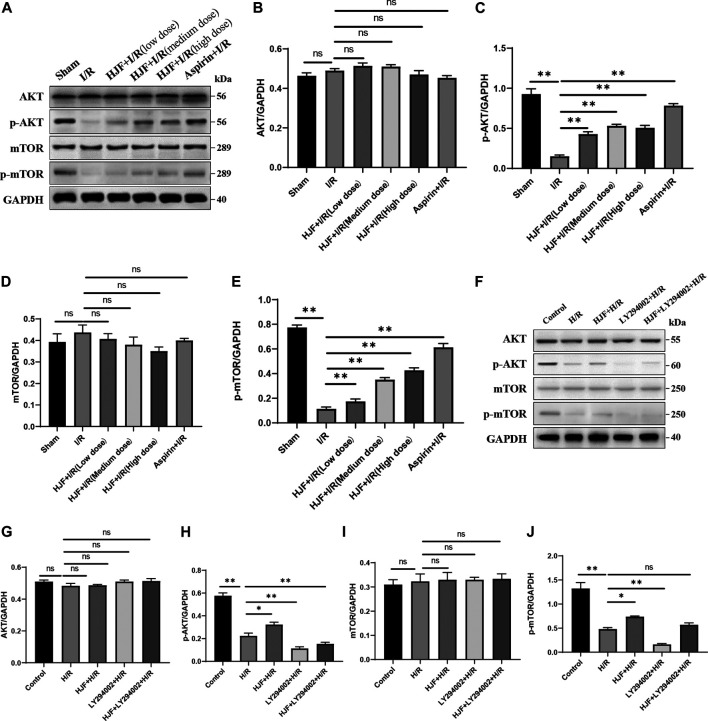
Effects of HJF on the PI3K/AKT/mTOR signalling pathway. **(A)**, Representative western blot images of AKT and mTOR in rats; **(B)**, Quantification of the levels of AKT in rat hearts; **(C)**, Quantification of the levels of *p*-AKT in rat hearts; **(D)**, Quantification of the levels of mTOR in rat hearts; **(E)**, Quantification of the levels of *p*-mTOR in rat hearts; **(F)**, Representative western blot images of AKT and mTOR in H9C2 cells; **(G)**, Quantification of the levels of AKT in H9C2 cells; **(H)**, Quantification of the levels of *p*-AKT in H9C2 cells; **(I)**, Quantification of the levels of mTOR in H9C2 cells; **(J)**, Quantification of the levels of *p*-mTOR in H9C2 cells. The data are shown as the means ± SDs in each group (n = 3). *p < 0.05, **p < 0.01. I/R, ischaemia/reperfusion; H/R, hypoxia/reoxygenation; HJF, Huoxue Jiedu Formula.

According to the results of network pharmacology and the literature review, the PI3K/AKT/mTOR pathway is closely associated with autophagy. Thus, the effects of HJF on the PI3K/AKT/mTOR signalling pathway were evaluated. The results demonstrated that compared with I/R group, pretreatment of HJF increased the phosphorylation of AKT and mTOR in mice (Figures 7A–E). In addition, compared with the H/R group, the HJF group showed the increased phosphorylation of AKT and mTOR (Figures 7F–J). In addition, LY290002, the inhibitor of the PI3K/AKT signalling pathway, offset the effects of HJF on the cardiac autophagy induced by H/R (Figure 4). Therefore, pretreatment of HJF may alleviate cardiac autophagy via activating PI3K/AKT/mTOR signalling pathway.

## Discussion

In the current study, the effects of HJF pretreatment on myocardial injury and cardiac autophagy were evaluated. We found that pretreatment of HJF decreased the myocardial infarct size, myocardial injury marker (CK-MB and cTnT) levels and cardiac autophagic flux in I/R rat model. In an *in vivo* study, pretreatment of HJF significantly decreased the H/R-induced autophagy in H9C2 cells. Then, network pharmacology was used to predict the possible mechanism by which HJF regulates cardiac autophagy, and the PI3K/AKT/mTOR signalling pathway was the most significantly enriched pathway. In addition, experimental studies also demonstrated that pretreatment of HJF increased the phosphorylation of AKT and mTOR, while the effects were offset by PI3K inhibitor LY294002, which specifically blocks the PI3K/AKT pathway. Therefore, pretreatment of HJF alleviated myocardial injury and excessive autophagy by activating PI3K/AKT/mTOR signalling pathway. In summary, we revealed a novel mechanism by which pretreatment of HJF efficiently inhibited the excessive autophagy induced by I/R. To best of our knowledge, this is the first study to demonstrate the beneficial effects of HJF in preventing against myocardial I/R injury by decreasing excessive autophagy through activating of the PI3K/AKT/mTOR signalling pathway.

The role of autophagy in cardiomyocytes is associated with the degree and duration of stress (Meyer et al., 2013). Under physiological conditions, autophagy is cytoprotective, and contributes to cardiomyocyte recovery after transitory myocardial ischaemia. However, long-term reperfusion leads to excessive autophagy, which contributes to the excessive degradation and self-digestion of cardiomyocytes (Kini and Singh, 2007; Matsui et al., 2007; Sadoshima, 2008). Many studies have demonstrated that excessive autophagy induced by reperfusion leads to irreversible damage of cardiomyocytes and, ultimately, cell death (Matsui et al., 2007; Sciarretta et al., 2011). In addition, previous studies have demonstrated that decreasing excessive cardiac autophagy could remarkably improve cardiac function and alleviate myocardial injury after myocardial I/R in both *in vivo* and *in vitro* models (Yao et al., 2015; Fan et al., 2016; Huang et al., 2017; Zheng et al., 2017). A similar effect was observed in the present study. In our study, pretreatment of HJF decreased the myocardial infarct size and myocardial injury marker (CK-MB and cTnT) levels in rats subjected to I/R, and the effects may be attributed to decreases in I/R-induced autophagy. In an *in vitro* study, we found that pretreatment of HJF decreased H/R induced autophagy in H9C2 cells, which was consistent with animal experiments.

Based on the network pharmacology results and literature reviews, PI3K/AKT/mTOR signalling pathway plays an essential part in regulating cardiac autophagy. mTOR, a gene regulating cell growth, was suggested to decrease autophagic activity (Jung et al., 2010; Lekli et al., 2017; Saxton and Sabatini, 2017). And previous studies have revealed that AKT has cardioprotective effect after I/R or H/R (Jiang et al., 2016; Shu et al., 2017; Zhang et al., 2017). Our data showed that pretreatment of HJF increased the phosphorylation of AKT and mTOR in rats subjected to I/R. Consistent with vivo experiments, the results of vitro experiments revealed that pretreatment of HJF increased the phosphorylation of AKT and mTOR in H9C2 cells treated with H/R, and the effects were notably abolished by LY294002 which specifically blocks the PI3K/AKT pathway.

In conclusion, we demonstrated that pretreatment of HJF protected against myocardial I/R injury by inhibiting excessive autophagy via activation of the PI3K/AKT/mTOR pathway *in vivo* and *in vitro*. This study will provide a new strategy to prevent I/R-induced myocardial injury.

## Data Availability

The original contributions presented in the study are included in the article/Supplementary Material, further inquiries can be directed to the corresponding authors.

## Abbreviations

I/R, ischemia/reperfusion; H/R, hypoxia/reoxygenation; ROS, reactive oxygen species; QXJYG, Qing-Xin-Jie-Yu granule; HJF, huoxue jiedu formula; CCK-8, cell counting kit-8; OD, optical density; DMEM, Dulbecco’s minimum essential medium; FBS, fetal bovine serum; PLP, *Paeonia lactiflora* Pall; LS, *Ligusticum striatum* DC; CTW, *Coptis teeta* Wall; CK-MB, creatine kinase-MB; cTnT, cardiac troponin T; EDTA, ethylenediaminetetraacetic acid; PVDF, poly vinylidene fluoride; cDNA, complementary DNA; OMIM, online mendelian inheritance in man; SD, standard deviation.
